# Developing and identifying the psychometric properties of empty nest syndrome SCALE

**DOI:** 10.1186/s40359-025-03064-6

**Published:** 2025-07-07

**Authors:** Maryam Ahmadi Khatir, Mahnaz Modanloo, Ali Dadgari, Homeira Khoddam, Leila Teymouri Yeganeh

**Affiliations:** 1 Golestan University of Medical Sciences, Gorgan, Iran, Islamic Republic of; 2 Shahroud University of Medical Sciences, Shahrud, Iran, Islamic Republic of

**Keywords:** Empty nest, Psychometry, Questionnaire, Aged

## Abstract

**Background:**

The parents’ reaction to children leaving home is called Empty Nest Syndrome. Given this syndrome’s cultural nature, this research aims to design and determine the psychometric properties of a scale to measure it based on its multiple stages.

**Methods:**

In this methodological study, Waltz’s (2010) four-phase approach was utilized to design the instrument for measuring ENS. The psychometric process involved determining the instrument’s validity and reliability. The face and the content validity were assessed; then, for construct validity, 550 eligible participants were involved. The data was analyzed using SPSS22 and AMOS24 software. Internal consistency reliability was measured using Cronbach’s alpha in two phases, and McDonald’s omega coefficient was calculated. To assess reliability (test-retest and ICC), 50 participants were involved in a two-week interval.

**Results:**

In the initial phase, 309 items were extracted using a five-point Likert scale. The psychometric phases calculated item impact scores ranging from 1.28 to 5, CVR from 0.47 to 0.87, and CVI from 0.68 to 1. The KMO (0.939) indicated sample adequacy, and Bartlett’s test showed a model fit for factor analysis. The EFA revealed five factors, reducing the item count to 37. After naming the factors, CFA examined relationships between observed and latent variables. The results confirmed the convergent and divergent validity of the scale.

**Conclusion:**

Based on its stages, the ENS scale contributes to understanding the syndrome among healthcare providers, enabling precise diagnosis and prevention of related issues. The scale possesses all the characteristics of a standard instrument, with adequate validity, reliability, and ease of scoring.

**Backgrou**

According to family development theory, a significant stage of growth occurs in the life cycle when adult children leave the parental home [1]. This stage is termed the Empty Nest (EN), which happens when the last child departs for reasons such as employment, education, or marriage, leaving the parents alone [2, 3]. Empty Nest Syndrome (ENS) refers to the various effects experienced by parents when an EN occurs. This stage typically coincides with midlife or older age for parents, each facing distinct challenges that can lead to more pronounced adverse effects of ENS in some individuals [4]. Much of the existing literature associates ENS with feelings of depression, anxiety, and loneliness [5–8]. However, the results of a hybrid study indicated that ENS encompasses a range of behaviors and reactions by parents to their children’s departure, rather than being limited solely to negative symptoms and consequences, which typically last no more than two years [3].

Overall, ENS may signify the first significant social loss and serve as a turning point into old age for some parents [9]. Conversely, for others, it can represent a period where individual freedom to pursue personal interests increases, leading to greater life satisfaction [10]. Various adverse conditions associated with ENS include health issues, reduced functional capacity, and psychosocial problems such as suicidal thoughts, changes in social support, economic difficulties, and physical dysfunction [11, 12].

Given the decreasing number of children in modern families and the growing aging population along with its associated complications, it is essential to focus on the quality of life for middle-aged and elderly individuals in order to develop effective programs aimed at improving their health. Therefore, limited resources necessitate the development of coping strategies for the challenges posed by an aging population, which represents a significant burden on society. Identifying and predicting behavioral patterns in developmental situations, including ENS, coupled with appropriate screening, can help reduce healthcare costs and underscore the urgent need for effective interventions by policymakers and health service planners.

It is essential to recognize that lifestyle patterns, ENS, and societal responses to it are shaped by a community’s social, cultural, and climatic conditions, which can present various opportunities or threats to middle-aged and elderly populations. Thus, providing standardized tools for measuring ENS could serve as a valuable resource for the timely identification and prevention of widespread resultant problems. In health and care organizations, one of the key factors for achieving success is the satisfaction of service recipients, influenced by various economic factors, costs, effectiveness, and methods of program delivery and protocols [13]. Therefore, employing cost-effective strategies for identifying and assessing issues is a priority for healthcare organizations.

Considering the importance of the ENS construct and the absence of an appropriate instrument for the assessment of Empty Nest Syndrome and its associated stages with sufficient validity and reliability, the investigator was inspired to undertake a research study focused on the development and evaluation of the psychometric properties of a novel ENS scale.

## Methods

### Study design

This methodological study used Waltz’s method in Iran from 2022 to 2023. The four steps of this method are choosing the conceptual model of the measurement process, determining the objectives of the measurement, developing a blueprint, and developing the instrument [14].

1-Select of a conceptual model: This study uses a mixed conceptual model (inductive-deductive); the findings were taken from the comprehensive literature review and the experiences of the participants in our concept analysis study [3].

2-Determination of the objectives of the measurement: Using the integration and comparison of the definitions of the theoretical stages and the fieldwork in our concept analysis study, the following objectives were extracted for the design of the questionnaire:

- Measuring the ENS.
-Determining the type of ENS (pathologic or normal).
-Determining the stage of ENS that the person is in.
- Determining the person’s behavior at each stage.

3-Development of a blueprint: The appropriate number of items for each measurement objective was determined in this step.

4-Development of the instrument: A large pool of 309 items was created, including 292 items derived from the final analysis phase of our hybrid study [3] and 17 items drawn from other questionnaire [15]. Following extensive reviews and revisions by the research team, 76 items were excluded, and 106 were amalgamated. Subsequently, 125 items progressed to the psychometric stage.

**Fig. 1:**
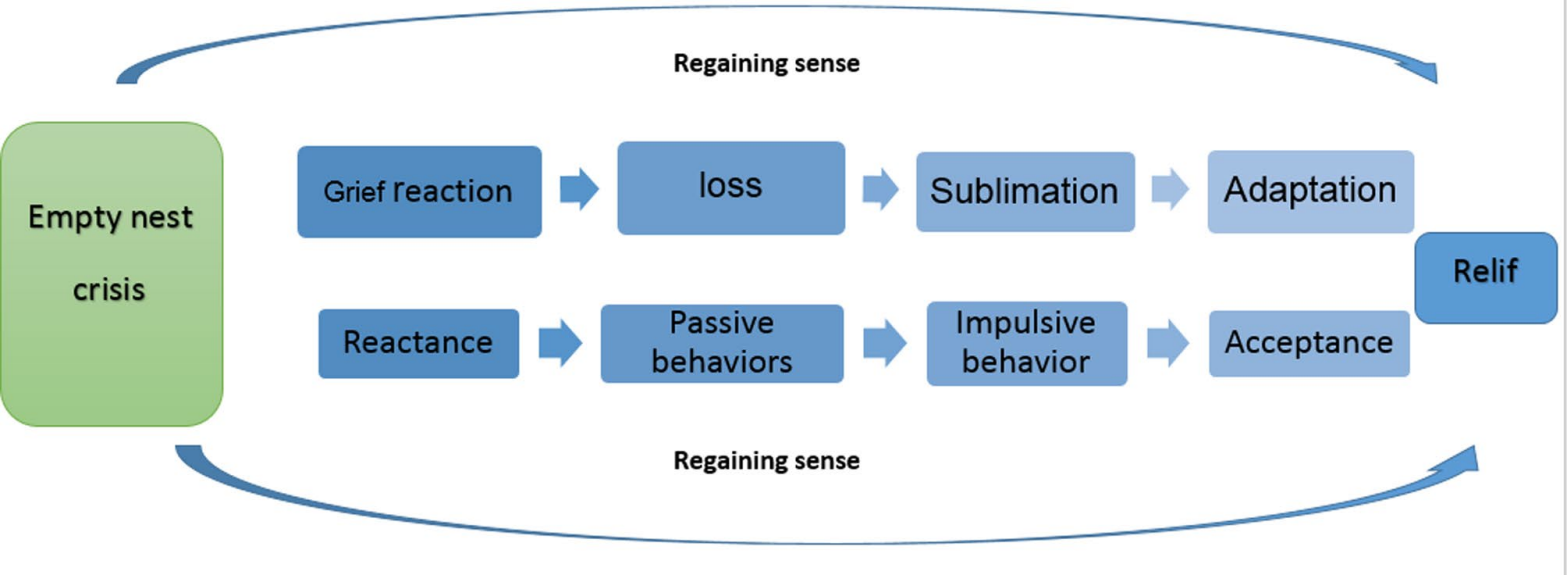
The process of ENS [3]

The final stage of our hybrid concept analysis studyproduced the results that established the ENS process (Fig. 1). This process represented the dimensions and main structures constituting the ENS concept and was the basis for developing the scale.

### Study sites and participants

The study setting for psychometric evaluation consisted of Comprehensive Healthcare Centers and Elderly Day Care Centers in Golestan, Iran.The eligibility criteria for the study included middle-aged and elderly individuals (ages 45 and older) with at least one child who has moved out due to education, work, immigration, marriage, or independence. Participants must not have experienced a child’s death or placement in a nursing home, must be able to communicate in Persian, possess full cognitive awareness, and have no confirmed psychiatric disorders. Ultimately, a total of 790 eligible middle-aged and elderly individuals were enrolled to evaluate the tool for assessing empty nest syndrome in this population.

### Psychometric evaluation

Face validity, content validity, construct validity, and reliability were assessed to determine the psychometric properties of the ENS scale.

A scale based on the Likert five-point scale (ranging from 5: utterly true of me to 1: not at all true of me) was designed, and the psychometric properties of the items were assessed based on classical theory (validity and reliability) [14]. The items with positive meaning were reverse-scored during the grading process.

### Face validity evaluation

Face validity was evaluated using qualitative and quantitative methods. In qualitative evaluation, face-to-face interviews were conducted with 20 middle-aged and elderly participants selected through purposive sampling based on specific inclusion criteria. The research team collected and reviewed their comments on each questionnaire item’s difficulty, appropriateness, and ambiguity.

Then, the same of the 20 persons were asked to evaluate the quantitative face validity using a scoring impact technique with a 5-point Likert scale ranging from 1 (not important) to 5 (Completely important). Items that received an importance score of 1.5 or higher were retained in the questionnaire. The average importance score for each item was calculated using the formula [14]:

Impact Score = Frequency (%) × Importance.

### Content validity evaluation

Content validity was evaluated through both qualitative and quantitative methods. In the qualitative evaluation, a group of fifteen experts in geriatric nursing(*n* = 4),gerontology(*n* = 3), psychology(*n* = 3),psychiatry(*n* = 1), and instrument development(*n* = 4) reviewed the grammar, wording, allocation, and scaling of the items. After receiving their comments, the items were adjusted.

In the quantitative aspect, the Content Validity Ratio (CVR) and Content Validity Index (CVI) were calculated. The same 15 experts rated items essentiality on a three-point scale(1: Not essential, 2: Useful but not essential, 3: Essential) [16], and CVR was calculated using the (ne-N/2)/(N/2) formula(neis the number of experts rates the items essential and N is the total number of experts [17]. The minimum acceptable CVR value was 0.49 based on Lawshe’s critical value [18]. CVI scores were determined for each item and the entire questionnaire. Experts used a four-point Likert scale to assess the “relevance” of each item. Scale Content Validity Index (S-CVI) was obtained by calculating Item Content Validity Index (I-CVI) values, and the average I-CVI for all items was computed based on expert ratings [16].

The Scale Content Validity Index (S-CVI) was calculated by first determining the Item Content Validity Index (I-CVI) for each item in the instrument, followed by averaging the I-CVI values for all items. Items with a CVI below 0.70 were eliminated.- Modified Kappa values were computed for each item to address chance effects. Items with Kappa scores falling between 0.60 and 0.74 were considered good, while those exceeding 0.74 were deemed excellent. All items met acceptable CVI and Kappa criteria [19].

### Construct validity evaluation

Construct validity was evaluated using exploratory factor analysis(EFA), convergent and divergent validity, and confirmatory factor analysis(EFA), which were used to investigate the causal relationships between the observed variables (questionnaire items) and hidden variables of data interpretation [20]. The sample size comprised 550 middle-aged and elderly individuals, with 300 participants allocated for Exploratory Factor Analysis (EFA) and 250 for Confirmatory Factor Analysis (CFA) [21]. The sample was randomly divided into two equal groups, where EFA was performed on one subsample and CFA on the other.

The participants completed the created questionnaire within 6 months. First, all eligible people were considered as the target group, and then they were selected randomly using a table of random numbers and entered the study. After completing the questionnaires, the data was analyzed using SPSS22 and AMOS24 software.

### Exploratory factor Analysis(EFA)

Williams et al. (2010) proposed a five-step protocol for performing Exploratory Factor Analysis (EFA), which involves assessing data suitability, extracting factors from the data, determining factor extraction criteria, selecting rotational methods, and interpreting results for construct labeling [22].

1-Data Suitability for Factor Analysis: The suitability of the data for factor analysis was assessed using the Kaiser-Meyer-Olkin (KMO) test and Bartlett’s Test of Sphericity (BT). KMO values falling within the range of 0.7 to 0.8 were deemed acceptable, while BT values ranging from 0.8 to 0.9 were considered excellent [23].

2-Factor Extraction Methods: Factors were extracted from the data using Principal Component Analysis and Maximum Likelihood Estimation methods.

3-Criteria for Factor Extraction: The number of factors to be extracted was determined based on the eigenvalue, scree plot, and cumulative variance percentage.

4-Rotational Method Selection: Varimax rotation simplifies and interprets the extracted factor structure.

5-Interpretation and Factor \Labeling: Each item in this study was retained at a minimum factor loading threshold of 0.5. The extracted factors were named according to the items comprising each factor, followed by an evaluation of their alignment with the concepts and dimensions identified during the qualitative stage.

### Confirmatory factor Analysis(CFA)

CFA was used to assess a model with predefined thresholds, employing maximum likelihood (ML)estimation on 250 samples. The assumption of ML is that indicators follow a normal distribution. ML is a crucial method in CFA estimation [24].

The model fit was examined with Root Mean Square of Error of Approximation [RMSEA < 0.08), Comparative Fit Index (CFI > 0.9), Goodness of Fit Index (GFI > 0.9), Adjusted Goodness of Fit Index (AGFI > 0.9), Normed Fit Index (NFI > 0.9), Parsimonious Normed Fit Index(PNFI > 0.5) and Incremental Fit Index (IFI > 0.9 were accepted [24].

### Convergent and discriminant validity

Convergent and discriminant validity assessment was conducted based on the Fornell-Larcker criterion with a sample size 300 [25]. Sampling was done concurrently with exploratory factor analysis from February to May 2023.

### Reliability

Cronbach’s alpha and McDonald’s omega coefficients were utilized for internal consistency measurement [19]. The Cronbach’s alpha coefficient was calculated in two stages: first, before construct validity, the alpha coefficient of the questionnaire was computed with a sample of 50 middle-aged and elderly individuals residing in empty-nest homes. In the second stage, after the factor analysis implementation, Cronbach’s alpha was calculated for the entire questionnaire and each factor with 120 participants. Subsequently, the omega coefficient was calculated.

To assess stability reliability using the Test-Retest method, 50 randomly selected participants who had not previously participated in the validity or reliability assessment completed a two-week interval test, which was determined through simple random sampling. After collecting data from both test periods, the Intraclass Correlation Coefficient (ICC) was calculated for the sub-scales and the entire questionnaire [26].

### Using, scoring, and interpreting assessment instruments

To ensure a better understanding and comparability of scores across different subscales of the questionnaire, a standardization method of 100 was used [27]. Higher scores indicate more severe ENS symptoms, with total scores interpreted as mild, moderate, or severe. The average completion time of the questionnaire was also assessed to evaluate the ease of tool use. The COSMIN^1^ checklist was utilized to assess the psychometric properties in this study [28].

## Results

The majority of participants in the present study were female (51.39%) and belonged to the age group of 54–65 years. Additional demographic characteristics are presented in Table 1.

**Table 1:** Demographic characteristics of participants in the evaluation of the ENS scale

Demographic Characteristic	Category	Frequency (*n*)	Percentage (%)
Gender	Male	384	48.61
	Female	406	51.39
Age (years)	45–54	193	31.6
	54–65	376	38.0
	65≤	221	30.4
Marital Status	Married	556	70.38
	Widowed	143	18.10
	Divorced	91	11.52
Education Level	No formal education	32	4.05
	Primary education	298	37.72
	Secondary education	425	53.80
	Higher education	35	4.43
Income Level	Low	189	23.93
	Middle	456	57.72
	High	145	18.35
Chronic Illness	Yes	308	38.99
	No	482	61.01
Number of Children	1	259	32.78
	2	306	38.74
	3≤	228	28.48
Time of Child’s Departure(Month)	6–12	421	53.29
	12–18	254	32.15
	18–24	92	11.65
	24–30	23	2.91
Living Area	Urban	438	55.44
	Rural	352	44.56

### Face and content validity

Most questionnaire items were revised in the qualitative face validity stage to enhance participants’ comprehension and clarity. For quantitative face validity, 11 items with scores below 1.5 were removed. Based on expert feedback, the wording of some items was adjusted in the qualitative content validity phase, resulting in 53 items being merged and transformed into 15 items due to overlap. The number of items was reduced from 114 to 76. After the Content Validity Ratio (CVR) calculation, 19 items with scores below 0.49 were excluded. The remaining 57 items underwent content validity index (CVI) and modified Kappa coefficient assessment.

Additionally, ten items with CVI scores below 0.70 were eliminated. Kappa coefficients were computed for each item, all scoring above 0.74, thus being retained for the subsequent stage. Consequently, the item count decreased from 57 to 47. The Scale Content Validity Index (S-CVI) was determined to be 0.865.

## Construct validity

### Exploratory factors analysis

With a value of 0.939, the KMO was higher than the recommended minimum of 0.6 (Kaiser, 1974). This indicates that the data in this study is suitable for analysis. The test of Sphericity was highly significant with χ2 = 193.8970 and *p* < 0.000. The Bartlett Sphericity test’s significance suggests a sufficient correlation between variables for factor analysis. Therefore, these results supported the data as appropriate for factor analysis. .

**Fig. 2:**
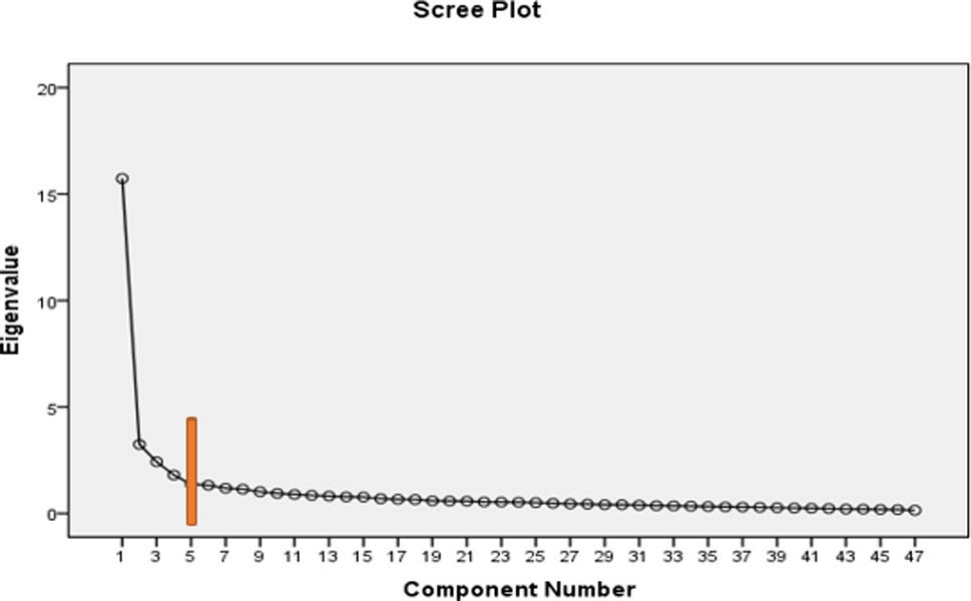
Scree plot of eigenvalues of the extracted factors in the EFA

Factors with eigenvalues greater than 1.35 and those outside the horizontal line formed by scree plot breaks were considered in determining the number of questionnaire components. These factors were required to explain a minimum of 50% of the variance of the concept of interest [20]. Figure 2 suggests that five factors sufficiently explain the factor structure of the questionnaire, following an assessment of the internal consistency of items.

During the factor extraction stage, variables with high intercorrelations were grouped into single factors. A factor loading of at least 0.5 was set as the threshold for this study. Table 2 shows the variance explained by each factor before and after rotation and the total variance explained by the five extracted factors. The table also highlights identifying five factors with eigenvalues exceeding 1.5, collectively explaining 82.3% of the variance. The cumulative percentage of variance accounted for by the remaining 42 factors was 17.7%. Five factors were identified from the initial EFA for the 47 items with eigenvalues greater than 1.35. The scree plot also showed that five factors were appropriate (Fig. 2).

**Table 2:** Factor analysis: total explained variance for the 5 factors of ENS scale

components	initial eigenvalues			Total squared factor loadings before rotation			Total squared factor loadings after rotation		
	Total	Percentage of variance	cumulative percentage	Total	Percentage of variance	cumulative percentage	Total	Percentage of variance	cumulative percentage
1	15.728	33.463	33.463	1.4285	33.463	33.463	7.350	15.637	15.637
2	3.246	6.907	40.370	3.246	26.907	60.370	5.224	21.115	36.752
3	2.428	5.167	45.537	2.428	5.167	65.537	4.946	10.524	47.276
4	1.806	3.843	49.380	1.806	3.000	68.537	4.595	19.777	67.053
5	1.376	2.928	52.308	1.376	12.928	81.465	2.470	15.255	82.308

Extraction Method: Principal Component Analysis

Rotation Method: Varimax with Kaiser Normalization

Five factors were identified based on the rotated factor matrix (Varimax rotation), collectively explaining 82.3% of the total variance, and were deemed acceptable.

- Factor 1 (12 items) includes items 1, 3, 5, 7, 8, 13, 21, 31, 33, 36, 38, 47.
- Factor 2 (16 items) includes items 2, 4, 9, 10, 11, 12, 14, 15, 16, 18, 19, 25, 28, 29, 41, 46.
- Factor 3 (8 items) includes items 20, 32, 34, 35, 39, 40, 44, 45.
- Factor 4 (6 items) includes items 17, 22, 23, 24, 30, 42.
- Factor 5 (5 items) includes items 6, 26, 27, 37, 43.

Ten items were removed at this stage, reducing the total scale to 37. After the factors had been formed, naming was done based on the results of our concept analysis study [3]. Therefore, the explanation of the tool’s expressions and their naming serves as the point of integration between the stages of ENS and the study, where the expressions constituting each factor are the same subcategories obtained from the conceptual analysis of ENS.

### Confirmatory factors analysis (CFA)

**Table 3:** Model fit indices

Index	Target Value	Reported Value
*χ*_2_	≥ 0.05	1539.45
p-value	≥ 0.05	0.07
df	-	569
*χ*_2_/*df*	≥ 3	2.705
GFI	≥ 0.9	0.952
AGFI	≥ 0.9	0.945
NFI	≥ 0.9	0.936
IFI	≥ 0.9	0.921
CFI	≥ 0.9	0.972
RMSEA	≥ 0.05	0.03

“See Table 4” ten items were removed at this stage, reducing the total scale to 37. After the factors had been formed, naming was done based on the results of our concept analysis study [3]. Therefore, the explanation of the tool’s expressions and their naming serves as the point of integration between the stages of ENS and the study, where the expressions constituting each factor are the same subcategories obtained from the conceptual analysis of ENS.

**Table 4:** ENS scale after exploratory factor analysis and item reduction

Factor Load	Subscales		Item No.	Items
2	The first phase of ENS	Mourning reaction	2	I feel that my life has become meaningless.
2			3	I feel worthless.
2			7	I blame myself for not being a good parent to my child/ren.
2			8	I often feel lonely for my child/ren.
2			9	I am restless and anxious.
2			11	I feel hopeless.
2			12	I often feel like crying.
2			13	I worry about the future.
2			15	Activities that used to bring me joy in the past are no longer enjoyable.
2			21	I suffer from loneliness.
2			24	I cannot sleep well.
2			34	My life is not fulfilling without my child/ren.
		Resistance reaction	16	My child/ren leaving does not matter to me.
2			6	I do not need anyone’s help in my life.
2			37	I want to live with my child/ren after marriage (independence).
1	The second phase of ENS	Feelings of loss	1	I feel I have lost my role as a parent.
1			4	I feel my child/ren has forgotten me.
1			30	I need to see my child/ren regularly.
1			32	I feel I have no one to rely on when facing problems after my child/ren has left.
1		Passive behaviors	10	I have become indifferent and apathetic towards issues around me.
1			26	I have neglected my health.
1			27	I do not care about my appearance after my child/ren has left.
4	The third phases of ENS	sublimation	18	I want to learn a new skill or pursue further education.
4			19	I want to meet new people.
4			35	I strive to adapt to new life circumstances.
4		Impulsive behaviors	20	It is hard for me to focus on tasks or my job after my child/ren has left.
4			25	Shopping gives me peace after my child/ren has left.
4			14	I quickly get angry about the past.
5	The fourth phase of ENS	acceptance	5	I can set goals for my life.
5			22	I am willing to live in a nursing home if necessary.
5			23	I do not interfere in my child/ren’s personal life.
5			31	Accepting the independence of my child/ren is challenging for me.
5			36	I enjoy the silence of the house.
3	The fifth phase of ENS	Relief	17	I feel more at ease after my child/ren has left.
3			28	Since my child/ren has left home, I focus more on my hobbies (traveling, leisure, reading, etc.).
3			29	I feel a sense of freedom and liberation.
3			33	Being alone (with my spouse) is a pleasant experience.

The convergent and discriminant validity of the questionnaire was assessed using a sample of 300 individuals in the exploratory factor analysis phase. The findings indicated that the questionnaire’s convergent validity was supported across all subscales, as evidenced by CR > AVE. Moreover, meeting the criteria of CR > 0.7 and AVE > 0.5 further confirmed the questionnaire’s convergent validity (Table 4). Additionally, the questionnaire demonstrated discriminant validity, with all subscales showing MSV < AVE and ASV < AVE, thus solidifying the questionnaire’s discriminant validity [29] (Table 5).

**Table 5:** Results of convergent and discriminant validity criteria of the ENS scale

Factor Number (Subscale)	Factor Name (Subscale)	AVE	MSV	ASV	CR	Result
1	The second stage of ENS	0.6123	0.5128	0.4384	0.7542	Valid
2	The first stage of ENS	0.5891	0.5294	0.3245	0.8451	Valid
3	The fifth stage of ENS	0.6245	0.4854	0.3894	0.7029	Valid
4	The third stage of ENS	0.7253	0.6423	0.5237	0.7461	Valid
5	The fourth stage of ENS	0.7189	0.6784	0.6037	0.8254	Valid
**Total Scale**		0.65402	0.56966	0.45594	0.77477	Valid

### Introduction and scoring of the scale

This study introduces the ENS Scale (ENSS), designed to gauge the severity of ENS symptoms and track the syndrome’s progression among individuals with EN. Comprising 37 items, the ENSS offers an instrument for clinical research applications. A standardized scoring scheme out of 100 was employed, where the scores from the five domains were aggregated and divided by 5 to yield a total score ranging from 0 to 100. A higher score signifies a more pronounced presentation of ENS symptoms. Interpretation categorizes total scores into mild, moderate, and severe severity levels (Table 6).

The ENS Scale offers insights into the pathological aspects of ENS and tracks the syndrome’s progression among parents whose children have vacated the family home. The average completion time for the ENS Scale is 7 min.

**Table 6:** Subscales and total score domains of the ENS scale

Factor Number (Subscale)	Factor Name (Subscale)	Minimum and maximum score	Final scoring is based on a total of 100
2	The first stage of ENS	15–75	mild symptoms ≤ 33.3 33.3 < moderate symptoms ≤ 66.6 severe symptoms > 66.6
1	The second stage of ENS	7–35	
4	The third stage of ENS	6–30	
5	The fourth stage of ENS	5–25	
3	The fifth stage of ENS	4–20	
**Total Scale**		**37–185**	**20–100**

## Discussion

The aim of designing and validating the instrument is to develop a reliable tool based on established theories of instrument development for conducting studies within the target population [30]. This tool will enable accurate measurement of the intended concept and facilitate the identification of existing differences within the studied population. Given the insufficiency of knowledge regarding the operationalization of the ENS concept and the absence of a specific instrument in this area, the researchers conducted this study to design and determine the psychometric characteristics of the ENS measurement tool. This effort aims to fill the knowledge gap by achieving a common understanding of the topic and identifying areas for further development.

While various existing instruments predominantly focus on the negative symptoms associated with ENS [15, 31, 32], our tool is distinct in that it also highlights the positive aspects of this experience. Furthermore, our target includes both middle-aged individuals and older adults. It is important to recognize that ENS can emerge during middle age, even before the aging process is fully underway. However, many current tools have overlooked this demographic concentrating mainly on older adults. This oversight restricts a comprehensive understanding of ENS and its implications across various stages of life.

The development of the ENSS is grounded in the stages of ENS identified through our conceptual analysis, ensuring that all essential dimensions of this multifaceted phenomenon are comprehensively addressed. By incorporating both negative and positive aspects of the experience, our dual-perspective approach facilitates a more holistic evaluation of ENS across different life stages.

Considering the complexity of the ENS concept, the designed questionnaire must encompass multiple dimensions and sub-scales to cover all relevant content.

In our hybrid study of concept analysis, the concept and dimensions of ENS were identified and defined [3]. The present study involved designing the tool based on the characteristics extracted from the ENS concept through the hybrid study, alongside a review of existing questionnaires and item pools.

This research evaluated various types of validity essential for the instrument. The ENS Scale (ENSS) demonstrated adequate face and content validity. An overall variance of 82.08% indicates the sufficiency of the factors extracted from the EFA, confirming the tool’s construct validity. Furthermore, based on the results of the CFA, the fit of the ENS factor model was validated according to model fit indices, indicating that the model structure aligns with the data obtained from the statistical population. Additionally, the tool’s convergent and discriminant validity were well established.

The internal consistency was calculated in two phases: before establishing construct validity and after determining construct validity. The reported McDonald’s omega values also indicated appropriate and acceptable internal consistency for the sub-scales and the questionnaire.

One concern regarding the designed instruments is their degree of stability across repeated measurements, which closely aligns with the definition of reliability. The tool’s stability in this study was examined through the test-retest method [30]. The retest would occur two weeks after the initial test to ensure that no significant change in the measured variable (ENS) occurred during that period and that participants would not remember their prior responses. Participants’ statements during the study supported this notion when they reported, “I do not remember what I answered last time.”

According to Terwee et al. (2007), repeatability, which includes intra-cluster correlation and agreement, is a necessary criterion for evaluating an instrument. The two-way random model is the preferred method for intra-cluster correlation, with a value above 0.80 indicating appropriate stability [33]. In this study, the repeatability of the questionnaire was assessed using the intra-cluster correlation index, with the lowest value being 0.815 for the third sub-scale and the highest being 0.855 for the fourth sub-scale; the overall repeatability of the questionnaire was confirmed with an intra-cluster correlation of 0.839.

The questionnaire’s scoring method was standardized to a scale of 100, allowing individual scores to be compared with those of others and enabling the examination of the effects of preventive interventions or interventions managing ENS.

The final format of the ENSS included five sub-scales, each assessing various stages of ENS. The first, second, and fourth sub-scales each contained two dimensions, with necessary explanations in the discussion section related to the stages of ENS.

The average completion time of 7 min for the questionnaire indicates that it is simple and easy for respondents to complete, which may encourage individuals to participate in research utilizing this tool.

The ENSS is a valid and reliable tool that has been designed and validated in the context of Iranian culture, which can be used to assess both the pathological and normal aspects of ENS and to explore the stages of the syndrome in individuals whose last child has left home. By identifying each individual’s score in each stage, it is possible to ascertain which stage and dimension of the syndrome the individual is experiencing and, if necessary, to implement the required interventions.

In this study, the design of the questionnaire operationalized the results obtained from the qualitative phase in our concept analysis study. The findings from EFA essentially confirmed the results from the qualitative component. The items’ suitable factor loadings, along with their correlations and the formation of factors that aligned well with the qualitative stages of the study, indicate that the quantitative findings can contribute to the generalizability of the qualitative results. However, to ensure adequate generalizability of the findings, conducting studies with larger sample sizes in diverse environments seems necessary. Additionally, future longitudinal studies are recommended to assess the predictive validity of the ENSS, particularly in identifying parents at risk of developing severe forms of ENS. Such studies would help determine the tool’s utility in early detection and timely intervention.

### Limitations and future directions

This study faced cultural and social limitations affecting the tool’s design, requiring adaptations for diversity and representativeness. Gender differences were not analyzed, which may impact factor structure and generalizability; future research should address this. Due to time and budget constraints, the natural duration of ENS stages was not examined and will be explored in future studies. Evaluating the tool across different regions remains essential.

## Conclusion

As far as researchers have examined, the designed instrument is the first questionnaire specifically intended to measure ENS based on the experiences and perceptions of middle-aged and elderly individuals in EN situations. This questionnaire not only identifies ENS in terms of its pathological or normal aspects but also assesses the stage in which an individual is located and the type of behavior exhibited during each stage. Simple scoring, ease of use, appropriate validity and reliability, and adaptability to different communities are among the advantages of the present questionnaire. Utilizing the findings from our concept analysis study has resulted in a good alignment of the designed tool with the context of Iran, and leveraging related instruments can somewhat enhance the generalizability of the tool to other communities. However, the characteristics of this instrument, like any other tool developed based on classical measurement theory, depend on the respondents, and its adaptation to new contexts is essential. Given the points above, it is hoped that this research has made a valuable contribution toward producing knowledge related to the ENS construct and its quantitative measurement. It is anticipated that through conducting this study and designing the ENS questionnaire, a step will be taken toward investigating this syndrome among individuals in the EN stage, thereby mitigating the irreparable consequences of this syndrome through timely and appropriate interventions.

## Data Availability

The datasets used and/or analyzed during the current study available from the corresponding author on reasonable request.

## Abbreviations

EN: Empty Nest
ENS: Empty Nest Syndrome
ENSS: Empty Nest Syndrome Scale
KMO: Kaiser-Meyer-Olkin
BT: Bartlett's Test of Sphericity
EFA: Exploratory Factor Analysis
CFA: Confirmatory Factor Analysis
CVI: Content Validity Index
I-CVI: Item Content Validity Index
S-CVI: Scale Content Validity Index
CVR: Content Validity Ratio
ML: Maximum Likelihood
RMSEA: Root Mean Square of Error of Approximation
NFI: Normed Fit Index
IFI: Incremental Fit Index
GFI: Goodness-of-Fit Index
AGFI: Adjusted Goodness of Fit Index
CFI: Comparative Fit Index
PNFI: Parsimonious Normed Fit Index
CR: Composite Reliability
AVE: Average Variance Extracted
ASV: Average Shared Variance
MSV: Maximum Shared Variance
ICC: Intraclass Correlation Coefficient
COSMIN: Consensus-based Standards for the selection of health Measurement Instruments

## Footnotes

1 Consensus-based Standards for the selection of health Measurement Instruments.
